# Breast cancer response to neoadjuvant chemotherapy: Pre-treatment NLR, PLR, LMR, and SII do not predict pathological complete response or residual cancer burden

**DOI:** 10.17305/bb.2026.14417

**Published:** 2026-06-23

**Authors:** İsmail Zihni, Mehmet Zafer Sabuncuoğlu, Burcu Zihni, Ebru Özan Sanhal, Veli Vural, Cumhur Arıcı

**Affiliations:** 1 Department of General Surgery, Akdeniz University Medical Faculty, Antalya, Türkiye; 2 Department of Cardiology, Antalya City Hospital, Antalya, Türkiye; 3 Department of Radiology, Akdeniz University Medical Faculty, Antalya, Türkiye

**Keywords:** Neutrophil-to-lymphocyte ratio, platelet-to-lymphocyte ratio, systemic immune-inflammation index, neoadjuvant chemotherapy, pathological complete response, residual cancer burden, breast cancer molecular subtype

## Abstract

Inflammation-based indices, derived from routine complete blood count parameters such as the neutrophil-to-lymphocyte ratio (NLR), platelet-to-lymphocyte ratio (PLR), lymphocyte-to-monocyte ratio (LMR), and systemic immune-inflammation index (SII), have garnered considerable attention as potential prognostic markers for treatment response in breast cancer. Nevertheless, their predictive utility in patients undergoing neoadjuvant chemotherapy (NACT) remains equivocal. This study aimed to investigate the association between pre-treatment inflammatory indices and pathological complete response (pCR) and residual cancer burden (RCB) in breast cancer patients receiving NACT. A retrospective analysis was conducted on 110 consecutive patients with invasive breast carcinoma who received NACT followed by curative surgery at a single institution. Pre-treatment values for NLR, PLR, LMR, and SII were calculated from routine laboratory data. Given the non-normal distribution of inflammatory indices, as determined by Shapiro–Wilk testing, non-parametric analyses were employed. Group comparisons utilized the Mann–Whitney *U* and Kruskal–Wallis tests. Predictive performance was assessed using receiver operating characteristic (ROC) analysis with 500-iteration bootstrap resampling. Multivariable logistic regression was applied to identify independent predictors of pCR. The findings revealed no significant associations between any of the inflammatory indices and pCR or RCB class distribution. ROC analyses indicated poor discriminatory performance for all indices, with area under the curve (AUC) values consistently approximating 0.50. In the multivariable analysis, only Ki-67 ≥14% independently predicted pCR (OR 7.077, 95% CI 2.210–22.662; *P ═* 0.001). In conclusion, pre-treatment NLR, PLR, LMR, and SII did not exhibit clinically meaningful predictive value for pathological response to NACT within this cohort. Tumor proliferative activity, as reflected by Ki-67, remained the most robust independent predictor of treatment response.

## Introduction

Over the past two decades, neoadjuvant chemotherapy (NACT) has become an integral part of breast cancer treatment, particularly for biologically aggressive subtypes such as human epidermal growth factor receptor 2 (HER2)-positive and triple-negative disease [1]. While its initial application primarily focused on improving surgical operability in locally advanced tumors, its role has significantly expanded in early-stage breast cancer management. Beyond tumor downstaging and facilitating breast-conserving surgery, NACT also enables a direct assessment of treatment sensitivity through postoperative pathological evaluation [1]. Given the persistent global increase in breast cancer incidence, with approximately 2.30 million new cases reported in 2022, identifying reliable predictors of treatment response remains a critical clinical objective in the neoadjuvant setting [2].

Pathological response following NACT is commonly assessed using pathological complete response (pCR) and the Residual Cancer Burden (RCB) classification system [3, 4]. The RCB model categorizes patients from RCB-0, signifying complete eradication of invasive disease, to RCB-III, indicating extensive residual tumor burden [3, 4]. Among these categories, pCR (ypT0/is ypN0) is generally considered the most favorable outcome, defined by the absence of residual invasive carcinoma in both the breast and axillary lymph nodes at surgery [5]. Previous studies, including the Collaborative Trials in Neoadjuvant Breast Cancer (CTNeoBC) pooled analysis, have demonstrated that achieving pCR is associated with improved long-term outcomes, particularly in HER2-positive and triple-negative breast cancer (TNBC) subtypes [5]. However, this prognostic effect appears less consistent in luminal tumors.

Despite ongoing advancements in systemic therapy, accurately predicting which patients will respond to neoadjuvant chemotherapy remains challenging. Conventional markers such as Ki-67, HER2 status, hormone receptor expression, and histological grade provide important prognostic information, yet their ability to predict individual treatment response in routine clinical practice remains limited [6, 7]. Consequently, increasing attention has shifted toward systemic inflammatory and immune-related biomarkers as potential tools for improving response prediction.

The rationale for investigating hematological inflammatory indices in breast cancer is rooted in the dynamic interplay between systemic immunity and the tumor microenvironment. Elevated circulating neutrophil and platelet counts relative to lymphocyte levels are generally indicative of a systemic pro-tumor and immunosuppressive state. Such an environment may impair effective cytotoxic immune surveillance and potentially reduce the extent of immunogenic cell death induced by anthracycline- and taxane-containing chemotherapy regimens [8, 9]. Among the commonly studied indices, NLR, PLR, LMR, and SII each represent distinct mathematical expressions of peripheral immune status. These parameters are calculated as follows: NLR = neutrophils/lymphocytes; PLR = platelets/lymphocytes; LMR = lymphocytes/monocytes; and SII ═ (platelets × neutrophils)/lymphocytes. A significant practical advantage of all four indices is their derivation directly from routine complete blood count measurements, obviating the need for additional laboratory testing or financial burden, thereby making them particularly attractive for widespread clinical use, especially in resource-limited settings.

Evidence regarding the Systemic Immune-Inflammation Index (SII) has been particularly notable. A meta-analytic evaluation demonstrated a significant association between elevated SII levels and poorer overall survival in breast cancer patients (hazard ratio [HR] 2.12; 95% confidence interval [CI] 1.61–2.79; *P <* 0.001) [8]. Moreover, at least one comparative study conducted in the neoadjuvant setting suggested that SII may outperform both NLR and PLR in prognostic discrimination [10]. In terms of pathological complete response prediction, Cullinane et al. pooled data from eight retrospective studies involving 1,586 patients and reported that lower pretreatment NLR values were significantly associated with higher odds of achieving pCR (OR 1.83; 95% CI 1.15–2.91; *P ═* 0.0003) [11]. Similar findings were later reinforced by a broader pooled analysis evaluating circulating inflammatory biomarkers, which demonstrated a pooled odds ratio (OR) of 1.66 (95% CI 1.32–2.09; *P <* 0.001) in favor of lower inflammatory burden [12].

More recently, a 2025 meta-analysis specifically focused on the Platelet-to-Lymphocyte Ratio (PLR), encompassing 24 studies and 7,557 patients, found that elevated PLR was significantly associated with a lower probability of pCR (HR 1.51; 95% CI 1.24–1.84; *P <* 0.0001) as well as poorer survival outcomes [13]. Data derived from individual cohorts have provided additional support for these observations. In a study including 95 patients, Yang and colleagues demonstrated significant associations between NLR, PLR, SII, and pCR status [14]. Similarly, Kaytaz Tekyol et al., in a Turkish cohort of 150 patients, reported that the predictive impact of inflammatory indices appeared to vary according to molecular subtype [15]. Nevertheless, despite the growing body of supportive evidence, negative or non-significant findings continue to be reported across the literature. Variability in cut-off definitions coupled with marked heterogeneity in molecular subtype composition among study populations has prevented these indices from being fully integrated into routine clinical practice.

The present study was designed to further clarify this issue within a well-defined consecutive patient cohort. Pretreatment NLR, PLR, LMR, and SII values were analyzed in relation to two pathological response outcomes—binary pCR status and ordinal RCB class distribution—among 110 breast cancer patients treated with neoadjuvant chemotherapy at a single institution. In addition, multivariable logistic regression analysis was performed to determine whether any of these inflammatory markers retained independent predictive significance after adjustment for established tumor biological characteristics.

## Materials and methods

### Study population and design

This retrospective cohort study utilized institutional data obtained from a single tertiary oncology center. Patients who underwent surgery between January 2020 and February 2026 were consecutively screened for eligibility. The study population comprised individuals with histopathologically confirmed invasive breast carcinoma who received NACT followed by curative-intent surgical treatment.

Four inclusion criteria were applied during patient selection: histological confirmation of invasive breast carcinoma; administration of at least one complete cycle of NACT prior to surgery; availability of a pre-treatment complete blood count obtained within 14 days before NACT initiation; and comprehensive pathological assessment of the surgical specimen, including assignment of a RCB class. Patients were excluded if they had a concurrent hematological malignancy, active systemic infection at the time of blood sampling, autoimmune disease, chronic systemic corticosteroid use, or incomplete clinicopathological records. Following the application of all eligibility criteria, 110 patients were included in the final analysis. Patients were screened consecutively over the study period, and all who satisfied the inclusion criteria without meeting any exclusion criterion were enrolled, yielding the final analytic cohort of 110. A patient-flow diagram detailing the numbers screened, excluded (with reasons), and finally included is provided as Figure 1. Of the 136 patients screened, 26 were excluded: 13 for missing receptor data (ER, PR, or HER2), 7 for an unavailable pre-treatment complete blood count, 3 for inaccessible external-center records, 2 for missing Ki-67 or histological grade, and 1 for recurrent disease that fell outside the study criteria.

**Figure 1.**
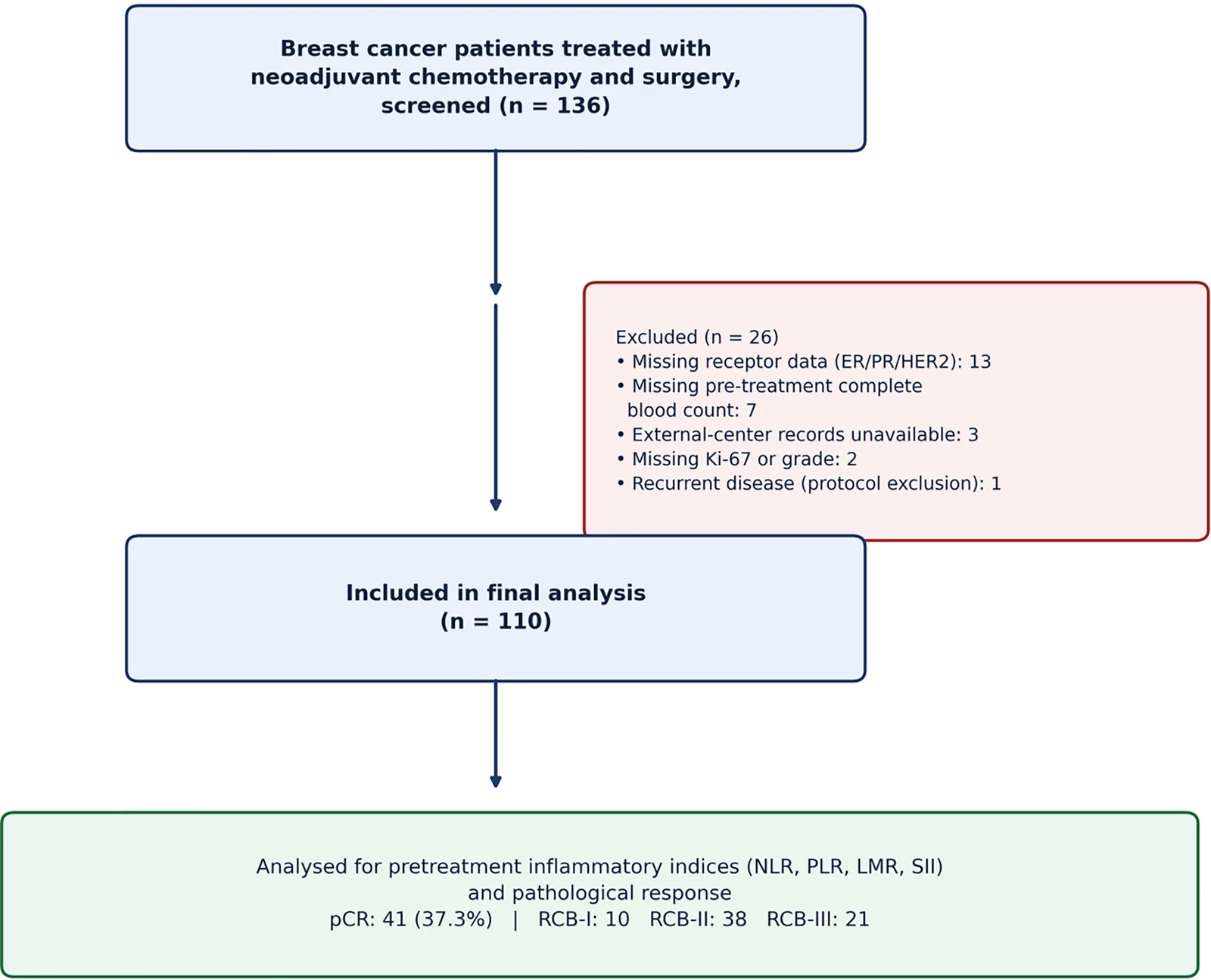
**Patient flow diagram.** Of 136 patients who received neoadjuvant chemotherapy and surgery during the study period, 26 were excluded, resulting in an analytic cohort of 110 patients. **Abbreviations:** ER, estrogen receptor; HER2, human epidermal growth factor receptor 2; LMR, lymphocyte-to-monocyte ratio; n, number of patients; NLR, neutrophil-to-lymphocyte ratio; pCR, pathological complete response; PLR, platelet-to-lymphocyte ratio; PR, progesterone receptor; RCB, residual cancer burden; SII, systemic immune-inflammation index.

The study protocol was approved by the local institutional ethics committee and conducted in accordance with the principles of the Declaration of Helsinki. All patient data were anonymized before statistical analysis, and no personally identifiable information was retained during the study process.

### Tumour characterization and molecular subtyping

Clinicopathological data were retrieved from the electronic medical records of patients who underwent surgery between January 2020 and February 2026. The collected variables included age at diagnosis, histological tumor type, clinical T and N stage, estrogen receptor (ER) status, progesterone receptor (PR) status, HER2 status, Ki-67 proliferative index, histological grade, and molecular subtype classification. Ki-67 was recorded only as a dichotomous variable using the established 14% threshold (<14% vs ≥14%); continuous Ki-67 values were not available in the retrospective records, which precluded analysis of Ki-67 as a continuous predictor or with alternative cut-offs.

Molecular subtypes were assigned according to the St. Gallen consensus criteria. Luminal A tumors were defined as ER/PR-positive, HER2-negative, and Ki-67 <14%. The Luminal B subtype included ER/PR-positive HER2-negative tumors with Ki-67 ≥14% as well as ER/PR-positive HER2-positive tumors. HER2-enriched tumors were characterized by ER/PR negativity together with HER2 positivity, whereas TNBCs were defined by the absence of ER, PR, and HER2 expression. Molecular subtypes were assigned using the St. Gallen surrogate definitions, with ER and PR positivity defined at the ≥1% threshold, HER2 positivity defined as immunohistochemistry (IHC) 3+ (or IHC 2+ with in situ hybridization [ISH] amplification), and a Ki-67 cut-off of 14%; tumors with low-positive hormone-receptor expression (1%–9%) were treated as receptor-positive in accordance with current guidelines. Receptor and subtype data were checked against the source records, and cases inconsistent with the stated surrogate definitions were reclassified. A complete cross-tabulation of ER, PR, HER2, Ki-67, and molecular subtype is provided as Table S1 to allow transparent verification of the classification.

ER and PR positivity were accepted when ≥1% nuclear immunoreactivity was detected on immunohistochemical evaluation. HER2 positivity was determined according to current American Society of Clinical Oncology/College of American Pathologists (ASCO/CAP) recommendations and was defined either by an immunohistochemistry score of 3+ or by confirmation of HER2 gene amplification using in situ hybridization methods.

### Derivation of inflammatory composite indices

Absolute neutrophil, lymphocyte, and monocyte counts, along with platelet counts, were obtained from pre-treatment complete blood count (CBC) samples collected nearest to the initiation of neoadjuvant chemotherapy. Inflammatory indices were calculated using the following formulas: Neutrophil-to-Lymphocyte Ratio (NLR) ═ absolute neutrophil count ÷ absolute lymphocyte count; PLR = platelet count ÷ absolute lymphocyte count; Lymphocyte-to-Monocyte Ratio (LMR) ═ absolute lymphocyte count ÷ absolute monocyte count; and SII ═ (platelet count × absolute neutrophil count) ÷ absolute lymphocyte count.

During data review, a single white blood cell (WBC) value of 568 × 10^9^/L was identified as biologically inconsistent with its corresponding differential count and was consequently excluded from analyses involving total WBC measurements. All CBCs were performed at the same accredited central laboratory using a standardized automated hematology analyzer. Patients with active systemic infection at the time of sampling were excluded *a priori* (refer to eligibility criteria). The flagged WBC value was determined to be a transcription error, incompatible with the patient’s differential count, and exclusively affected the total WBC field. The four inflammatory indices for this patient, derived from neutrophil, lymphocyte, monocyte, and platelet counts, were unaffected and therefore retained. Aside from this isolated outlier, all inflammatory index values were analyzed on their original continuous scale as recorded in the laboratory database, without winsorization or logarithmic transformation.

### Pathological response endpoints

The primary study endpoint was pCR, defined as the absence of residual invasive carcinoma in both breast tissue and regional lymph nodes at surgical resection (ypT0/is ypN0). Within the classification system proposed by Symmans et al. [4], this response corresponds to RCB class 0. RCB was calculated for each surgical specimen using the standard Residual Cancer Burden calculator (MD Anderson Cancer Center). This calculator integrates the two largest dimensions of the residual primary tumor bed, overall and *in-situ* cancer cellularity, the number of positive axillary lymph nodes, and the diameter of the largest nodal metastasis. Assessments were performed by the institution’s dedicated breast pathologists as part of routine diagnostic reporting, and the resulting continuous RCB index was used to assign RCB-0 to RCB-III classes.

The secondary endpoint was ordinal RCB classification. Patients were categorized into four groups: RCB-0, RCB-I, RCB-II, and RCB-III, representing a spectrum from complete pathological eradication to extensive residual macroscopic disease. The independent prognostic validity of this classification system has been previously demonstrated in a large multicenter pooled cohort comprising 5,161 patients [3].

### Ethics approval and consent to participate

The study was conducted in strict accordance with the ethical principles outlined in the Declaration of Helsinki. Approval was granted by the Non-Interventional Clinical Research Ethics Committee on March 26, 2026 (approval number 232). As the study was retrospective and based on de-identified records, the committee approved the analysis of clinical data collected between January 2020 and February 2026. The approval date of March 26, 2026, reflects the timing of the formal ethics application for this retrospective review and therefore postdates the data collection window. Given the retrospective design and the use of fully anonymized data, the requirement for written informed consent was waived by the committee.

### Statistical analysis

All statistical analyses were performed using Python 3.12. Core statistical and machine learning procedures were conducted with the SciPy 1.12 and scikit-learn 1.4 libraries. Prior to comparative analyses, distributional characteristics of continuous variables were assessed using the Shapiro–Wilk normality test. Given significant deviations from a normal distribution for the analyzed variables, the statistical workflow primarily employed non-parametric methods. Accordingly, non-parametric tests (Mann–Whitney *U* and Kruskal–Wallis) were used for all univariate group comparisons. Logistic regression and receiver operating characteristic (ROC) analysis were additionally applied for multivariable modeling and discrimination assessment, as detailed below.

Continuous variables are presented as median values with interquartile ranges (25th–75th percentile), while categorical variables are expressed as frequency and percentage. Comparisons between two independent groups for continuous variables were performed using the Mann–Whitney *U* test. For categorical data, Pearson’s chi-squared test was the primary method; Fisher’s exact test was used when expected cell counts were below five. Differences in inflammatory indices across the four RCB categories were evaluated using the Kruskal–Wallis H test.

The predictive performance of each inflammatory index for pathological complete response was examined through ROC curve analysis. Area under the curve (AUC) values, along with 95% confidence intervals, were estimated using non-parametric bootstrap resampling with 500 iterations. Optimal cut-off values for classification were determined according to the maximum Youden’s J statistic (J = sensitivity + specificity - 1).

For multivariable analysis, logistic regression modeling included NLR, PLR, SII, HER2 status, Ki-67 category (cut-off: 14%), histological grade (Grade 3 vs Grades 1–2), ER status, and age as a continuous covariate. Odds ratios and corresponding 95% confidence intervals were calculated based on Wald statistics. Statistical significance was defined as a two-sided *P*-value below 0.05 across all analyses. To maintain consistency with current clinical evidence, preference was given to literature published after 2020. LMR was not included in the primary adjusted model to limit the number of correlated blood-count-derived predictors, preserve the events-per-variable ratio, and because it exhibited no univariate association with pCR (Mann–Whitney U, *P ═* 0.77). To confirm that this choice did not affect the conclusions, a sensitivity model additionally including LMR was fitted (Table S2); LMR was not associated with pCR (OR 0.93, 95% CI 0.76–1.15, *P ═* 0.53), and Ki-67 remained the sole independent predictor. The primary multivariable model therefore evaluated NLR, PLR, and SII as the inflammatory predictors, and the manuscript’s wording reflects this.

Before fitting the multivariable model, collinearity among the inflammatory indices was examined using a Spearman correlation matrix and variance inflation factors (VIF). Given the right-skewed nature of the indices, a sensitivity analysis was conducted in which NLR, PLR, and SII were natural-log-transformed prior to entry into the logistic model. The discriminatory performance of the fitted multivariable model was quantified using the c-statistic (the area under the ROC curve of the model-predicted probabilities). With 41 pCR events, the events-per-variable ratio for the eight-covariate model was approximately five. To assess the stability of the estimates under this borderline ratio, a more parsimonious model retaining a single inflammatory index along with the clinicopathological covariates was additionally fitted.

In addition to data-derived thresholds, two externally established cut-offs (NLR ≥ 2.74 and SII ≥ 547) were applied to the present cohort and tested against pCR. The relationship between the inflammatory indices and the ordinal RCB outcome was further examined using an ordinal (proportional-odds) logistic regression model, complementing the Kruskal–Wallis analysis. No formal *a priori* sample-size calculation was performed; the cohort comprised all consecutive eligible patients treated during the study period. A *post hoc* consideration of statistical power is provided in the Discussion. As the four component blood-count values (neutrophil, lymphocyte, monocyte, and platelet counts) were available for every patient, all four inflammatory indices were computable for the entire cohort. Analyses were therefore performed on complete data for all 110 patients, requiring no imputation.

## Results

### Cohort characteristics and pCR distribution

The study cohort comprised 110 female patients, with a median age of 50.0 years (interquartile range [IQR]: 43.3–62.8). Molecular subtyping revealed the following distribution: Luminal A (*n* ═ 28, 25.5%), Luminal B (*n* ═ 41, 37.3%), HER2-enriched (*n* ═ 12, 10.9%), and TNBC (*n* ═ 29, 26.4%). HER2 positivity was observed in 36 patients (32.7%), and a Ki-67 proliferative index ≥14% was present in 67 patients (60.9%). Regarding tumor burden at diagnosis, 89 patients (80.9%) presented with clinical T1/T2 disease, while 69 patients (62.7%) were staged as cN0/N1. Baseline clinicopathological characteristics, stratified by pCR status, are detailed in Table 1.

**Table 1:** Baseline clinicopathological characteristics by pathological response

**Characteristic**	**Total (*n* ═ 110)**	**pCR (*n* ═ 41)**	**Non-pCR (*n* ═ 69)**	***P* value**
Age, median (IQR), years	50.0 (43.3–62.8)	49.0 (42.0–57.0)	51.0 (44.0–64.0)	0.292 ‡
**Molecular Subtype, *n* (%)**				
Luminal A	28 (25.5)	2 (4.9)	26 (37.7)	
Luminal B	41 (37.3)	21 (51.2)	20 (29.0)	0.107 †
HER2-enriched	12 (10.9)	8 (19.5)	4 (5.8)	
Triple-negative	29 (26.4)	10 (24.4)	19 (27.5)	
**ER Status, *n* (%)**				
Negative	41 (37.3)	18 (43.9)	23 (33.3)	
Positive	69 (62.7)	23 (56.1)	46 (66.7)	0.366 †
**PR Status, *n* (%)**				
Negative	61 (55.5)	24 (58.5)	37 (53.6)	
Positive	49 (44.5)	17 (41.5)	32 (46.4)	0.614 †
**HER2 Status, *n* (%)**				
Negative	74 (67.3)	21 (51.2)	53 (76.8)	
Positive	36 (32.7)	20 (48.8)	16 (23.2)	0.011 †*
**KI-67 Index, *n* (%)**				
Low (<14%)	43 (39.1)	5 (12.2)	38 (55.1)	
High (≥14%)	67 (60.9)	36 (87.8)	31 (44.9)	<0.001 †*
**Histological Grade, *n* (%)**				
Grade 1	5 (4.5)	2 (4.9)	3 (4.3)	
Grade 2	67 (60.9)	18 (43.9)	49 (71.0)	0.015 †*
Grade 3	38 (34.5)	21 (51.2)	17 (24.6)	
**Clinical T Stage, *n* (%)**				
cT1/T2	89 (80.9)	35 (85.4)	54 (78.3)	
cT3/T4	21 (19.1)	6 (14.6)	15 (21.7)	0.506 †
**Clinical N Stage, *n* (%)**				
cN0/N1	69 (62.7)	29 (70.7)	40 (58.0)	
cN2/N3	41 (37.3)	12 (29.3)	29 (42.0)	0.257 †
**Haematological Parameters**				
Hemoglobin, g/dL	12.2 (11.4–13.5)	12.2 (11.0–13.5)	12.4 (11.5–13.4)	0.672 ‡
Platelet count ×10^9^/L	271.5 (229.0–311.5)	275.0 (239.0–315.0)	271.0 (224.0–309.0)	0.793 ‡
WBC ×10^9^/L	6.98 (5.46–8.88)	6.53 (5.37–7.95)	7.15 (5.63–9.43)	0.213 ‡
Lymphocyte ×10^9^/L	1.92 (1.46–2.53)	1.85 (1.34–2.50)	1.98 (1.50–2.54)	0.617 ‡
Neutrophil ×10^9^/L	3.99 (3.01–5.75)	3.93 (3.01–5.20)	4.06 (3.02–6.13)	0.431 ‡

Continuous data: Median (IQR); categorical data: n (%). † Pearson chi-squared test was used. Fisher’s exact test was used to compare histological grade when an expected cell count was <5. ‡ Mann-Whitney *U* test was used. *P < 0.05. **Abbreviations**: cN, clinical nodal stage; cT, clinical tumor stage; ER, estrogen receptor; HER2, human epidermal growth factor receptor 2; IQR, interquartile range; pCR, pathological complete response; PR, progesterone receptor; WBC, white blood cell.

A pathological complete response (pCR; RCB-0) was achieved in 41 patients, resulting in an overall pCR rate of 37.3%. Among the 69 patients with residual disease, 10 (9.1%) were classified as RCB-I, 38 (34.5%) as RCB-II, and 21 (19.1%) as RCB-III. Analysis by molecular subtype revealed pCR rates of 7.1% in Luminal A tumors, 51.2% in Luminal B, 66.7% in HER2-enriched disease, and 34.5% in TNBC.

Univariate analyses identified statistically significant associations between pCR and three clinicopathological variables. Patients with HER2-positive tumors exhibited a significantly higher pCR rate compared to HER2-negative cases (55.6% [20/36] vs. 28.4% [21/74]; χ^2^ = 6.532, *P ═* 0.011). Similarly, a Ki-67 value ≥14% was markedly overrepresented among patients achieving pCR (87.8% vs. 12.2%; χ^2^ = 18.098, *P <* 0.001). Histological grade also demonstrated a significant association, with Grade 3 tumors showing higher pCR rates compared to Grades 1–2 (55.3% [21/38] vs. 27.8% [20/72]; χ^2^ = 8.380, *P ═* 0.015). Conversely, no statistically significant relationships were identified for age, clinical T stage, clinical nodal stage, ER status, PR status, or molecular subtype when analyzed as a composite categorical variable.

Shapiro–Wilk normality testing demonstrated significant deviation from a normal distribution for all evaluated inflammatory indices: NLR (*W* ═ 0.706, *P <* 0.001), PLR (*W* ═ 0.774, *P <* 0.001), LMR (*W* ═ 0.770, *P <* 0.001), and SII (*W* ═ 0.787, *P <* 0.001). Age distribution also showed statistically significant non-normality (*W* ═ 0.974, *P ═* 0.027). These findings collectively supported the use of a non-parametric analytical approach throughout the study.

### Inflammatory index values by pathological response category

Pretreatment inflammatory index values stratified by pathological response outcome are summarized in Table 2. None of the evaluated inflammatory ratios demonstrated a statistically significant difference between patients who achieved pathological complete response and those with residual disease after treatment. Median NLR values were 2.12 (IQR 1.56–2.82) in the pCR group and 2.08 (IQR 1.64–2.97) in the non-pCR group (*U* ═ 1388.0, *P ═* 0.822). For PLR, median values were 144.4 (IQR 112.2–169.3) among patients with pCR and 135.3 (IQR 108.4–182.3) among those without pCR (*U* ═ 1407.0, *P ═* 0.926). Median LMR values were similarly close between the two groups, measuring 3.77 (IQR 2.70–4.54) in patients achieving pCR and 3.66 (IQR 2.80–4.50) in patients with residual disease (*U* ═ 1395.5, *P ═* 0.870). SII values were also comparable, with medians of 599.0 (IQR 407.0–788.7) in the pCR group and 581.5 (IQR 415.2–862.6) in the non-pCR group (*U* ═ 1362.0, *P ═* 0.725).

**Table 2:** Pretreatment inflammatory index values stratified by response outcome

**Index**	**Full cohort (*n* ═ 110) Median (IQR)**	**pCR (*n* ═ 41) Median (IQR)**	**Non-pCR (*n* ═ 69) Median (IQR)**	**U**	***P***
**NLR**	2.08 (1.59–2.95)	2.12 (1.56–2.82)	2.08 (1.64–2.97)	1388.0	0.822
**PLR**	141.1 (111.1–180.7)	144.4 (112.2–169.3)	135.3 (108.4–182.3)	1407.0	0.926
**LMR**	3.75 (2.76–4.51)	3.77 (2.70–4.54)	3.66 (2.80–4.50)	1395.5	0.870
**SII**	584.4 (407.6–860.2)	599.0 (407.0–788.7)	581.5 (415.2–862.6)	1362.0	0.725

Mann–Whitney *U* test was used. **Abbreviations**: IQR, interquartile range; LMR, lymphocyte-to-monocyte ratio; NLR, neutrophil-to-lymphocyte ratio; pCR, pathological complete response; PLR, platelet-to-lymphocyte ratio; SII, systemic immune-inflammation index.

No inflammatory marker demonstrated a stable or clinically meaningful directional association with pCR. PLR and NLR values were only minimally higher in patients achieving complete response, while SII values also showed a slight numerical elevation in the pCR group despite substantial overlap in distribution. Overall, the observed differences between groups were small in magnitude and did not approach statistical significance.

### Residual cancer burden class analysis

When inflammatory indices were evaluated simultaneously across all four RCB categories, no statistically significant differences were observed between classes, as shown in Table 3. Kruskal–Wallis testing with 3 degrees of freedom yielded the following results: NLR, *H* ═ 0.415 (*P ═* 0.937); PLR, *H* ═ 0.580 (*P ═* 0.901); LMR, *H* ═ 0.432 (*P ═* 0.934); and SII, *H* ═ 0.130 (*P ═* 0.988).

**Table 3:** Inflammatory index values across residual cancer burden classifications

**Index**	**RCB-0 (*n* ═ 41)**	**RCB-I (*n* ═ 10)**	**RCB-II (*n* ═ 38)**	**RCB-III (*n* ═ 21)**	**H**	***P***
NLR	2.12 (1.56–2.82)	2.06 (1.54–2.54)	2.10 (1.65–2.96)	1.98 (1.70–3.03)	0.415	0.937
PLR	144.4 (112.2–169.3)	128.9 (113.9–184.1)	143.5 (116.1–181.6)	129.6 (101.5–175.8)	0.580	0.901
LMR	3.77 (2.70–4.54)	3.88 (3.46–4.43)	3.75 (2.60–4.26)	3.66 (2.85–4.89)	0.432	0.934
SII	599.0 (407.0–788.7)	711.4 (394.0–839.6)	571.7 (429.0–858.8)	562.5 (388.4–972.3)	0.130	0.988

All values are reported as median (IQR). The H statistic was computed with 3 degrees of freedom. No post-hoc pairwise comparisons were conducted given omnibus non-significance. **Abbreviations**: IQR, interquartile range; LMR, lymphocyte-to-monocyte ratio; NLR, neutrophil-to-lymphocyte ratio; PLR, platelet-to-lymphocyte ratio; RCB, residual cancer burden; SII, systemic immune-inflammation index.

Examination of median values across RCB-0, RCB-I, RCB-II, and RCB-III categories also failed to demonstrate a consistent or progressive distribution pattern for any inflammatory ratio. Notably, the RCB-I subgroup (*n* ═ 10) exhibited the highest median SII value (711.4), despite representing the lowest residual disease burden among patients who did not achieve pCR. This pattern did not support the presence of a coherent monotonic relationship between inflammatory index levels and increasing residual cancer burden.

### Independent pCR predictors: Multivariable logistic regression

Adjusted associations between clinicopathological variables, inflammatory indices, and pCR are presented in Table 4. A total of eight covariates were simultaneously entered into the multivariable logistic regression model for the full cohort (*N* ═ 110): NLR, PLR, SII, HER2 status, Ki-67 category, histological grade, ER status, and age. Among these variables, only Ki-67 ≥14% remained statistically significant in the adjusted analysis (OR 7.077; 95% CI 2.210–22.662; Wald *Z* ═ 3.276; *P ═* 0.001). This finding indicates that tumors with higher proliferative activity had more than a sevenfold increased likelihood of achieving pCR compared to low-proliferative tumors, even after adjustment for clinicopathological factors and the inflammatory indices included in the model (NLR, PLR, and SII).

**Table 4:** Multivariable logistic regression: Independent covariates associated with pCR

**Variable**	**OR**	**95% CI**	**Wald Z**	***P* value**
NLR (per unit)	1.165	0.806–1.683	0.813	0.416
PLR (per unit)	1.003	0.996–1.009	0.793	0.427
SII (per unit)	0.999	0.998–1.001	−1.021	0.308
HER2-positive	2.417	0.914–6.390	1.787	0.075
Ki-67 ≥14% (ref: <14%)	7.077	2.210–22.662	3.276	0.001 *
Histological grade 3	1.284	0.461–3.578	0.480	0.633
ER-negative	1.589	0.586–4.310	0.908	0.363
Age (per year)	0.980	0.942–1.019	−1.019	0.308

*P < 0.05. Reference categories were HER2-negative, Ki-67 <14%, Grade 1–2, and ER-positive. A complete-case analysis was performed (N=110). **Abbreviations**: CI, confidence interval; ER, estrogen receptor; HER2, human epidermal growth factor receptor 2; NLR, neutrophil-to-lymphocyte ratio; OR, odds ratio; pCR, pathological complete response; PLR, platelet-to-lymphocyte ratio; SII, systemic immune-inflammation index.

HER2 positivity demonstrated a tendency toward improved pCR rates; however, this association did not reach statistical significance (OR 2.417; 95% CI 0.914–6.390; *P ═* 0.075). In contrast, all inflammatory indices produced effect estimates close to unity. The calculated odds ratios were 1.165 for NLR (*P ═* 0.416), 1.003 for PLR (*P ═* 0.427), and 0.999 for SII (*P ═* 0.308). Confidence intervals for these parameters consistently crossed the null value, indicating a lack of independent predictive association between inflammatory index levels and pathological complete response. Collinearity diagnostics confirmed that the blood-count–derived indices were interrelated: NLR and SII were strongly correlated (Spearman ρ = 0.86), with variance inflation factors of 4.0 for NLR and 4.4 for SII, whereas PLR and LMR showed lower values (VIF <1.7). The fitted model discriminated moderately (c-statistic 0.79), a performance attributable almost entirely to Ki-67 and HER2 status rather than to the inflammatory indices. The findings were unchanged when NLR, PLR, and SII were natural-log–transformed (all *P* > 0.09) and when a parsimonious model retaining a single inflammatory index alongside the clinicopathological covariates was fitted; in every specification, Ki-67 ≥14% remained the only significant predictor of pCR and none of the inflammatory indices reached significance (Table S2).

### Receiver operating characteristic analysis

ROC curve analysis of inflammatory indices as binary pCR classifiers is presented in Table 5. ROC analysis demonstrated that the discriminatory performance of all four inflammatory indices remained close to the no-discrimination reference line. Bootstrap-based AUC estimates generated from 500 resampling iterations yielded the following results with corresponding 95% confidence intervals: NLR, 0.513 (0.374–0.596); PLR, 0.505 (0.406–0.615); LMR, 0.510 (0.368–0.594); and SII, 0.520 (0.363–0.594). None of the evaluated indices achieved an AUC value significantly exceeding 0.50. The consistently broad confidence intervals encompassing the null threshold further suggested that the lack of discriminatory capacity reflected a genuine characteristic of these markers within the present cohort rather than a consequence of limited sample size alone.

**Table 5:** ROC curve analysis: Inflammatory indices as binary pCR classifiers

**Index**	AUC (95% CI)*	**Cut-off†**	**Sensitivity**	**Specificity**	***P*‡**
NLR	0.513 (0.374–0.596)	≤1.62	36.6%	76.8%	0.822
PLR	0.505 (0.406–0.615)	≥111.0	82.9%	29.0%	0.926
LMR	0.510 (0.368–0.594)	≤4.09	73.2%	42.0%	0.870
SII	0.520 (0.363–0.594)	≤645.6	65.9%	46.4%	0.725

*500-iteration bootstrap resampling. †Youden’s J statistic (J = sensitivity + specificity − 1). ‡AUC vs. 0.50 by Mann–Whitney U. AUC, area under the curve. Cut-off values were determined by the maximum Youden index with pathological complete response coded as the positive outcome: Because the AUC approximated 0.50 for every index, these thresholds are exploratory and are not proposed as clinical decision cut-offs. ROC curves are provided in Figure S1. **Abbreviations**: AUC, area under the curve; CI, confidence interval; LMR, lymphocyte-to-monocyte ratio; NLR, neutrophil-to-lymphocyte ratio; pCR, pathological complete response; PLR, platelet-to-lymphocyte ratio; ROC, receiver operating characteristic; SII, systemic immune-inflammation index.

Cut-off values determined according to Youden’s J statistic also failed to produce clinically meaningful sensitivity–specificity profiles. For example, the NLR threshold (≤1.62) yielded a sensitivity of only 36.6% with a specificity of 76.8% (Table 5), a combination that would misclassify the majority of true responders. Such distributions offer limited practical relevance for treatment-selection or response-prediction purposes in routine clinical settings. Because the area under the curve approached 0.50 for every index, these Youden-derived thresholds are reported as exploratory only and are not proposed as clinical decision cut-offs. Applying two externally established thresholds to the present cohort produced the same null result: neither NLR ≥2.74 (pCR 33.3% vs 38.8%; *P ═* 0.763) nor SII ≥547 (pCR 36.1% vs 38.8%; *P ═* 0.925) was associated with pathological complete response. Consistently, an ordinal proportional-odds model relating the indices to RCB class showed no significant association (NLR *P ═* 0.42, PLR *P ═* 0.93, SII *P ═* 0.74), in agreement with the Kruskal–Wallis findings.

## Discussion

Four independent analytical approaches—binary comparison using the Mann–Whitney *U* test, ordinal RCB-category evaluation with the Kruskal–Wallis test, ROC-based discrimination analysis, and multivariable logistic regression modeling—consistently demonstrated that pretreatment NLR, PLR, LMR, and SII lacked measurable predictive value for pathological response to neoadjuvant chemotherapy in this breast cancer cohort. Inflammatory index distributions exhibited substantial overlap between patients achieving pCR and those with residual disease. Similarly, AUC values from ROC analysis remained only marginally above the no-discrimination reference threshold. None of the evaluated inflammatory ratios maintained statistical significance after multivariable adjustment.

In contrast, the Ki-67 proliferative index, reflecting intrinsic tumor biology rather than systemic immune-inflammatory status, emerged as the sole independent predictor of pCR. Highly proliferative tumors showed more than a sevenfold increase in response probability [6, 7]. The consistency of these negative findings across diverse analytical strategies reinforces the robustness of the overall conclusion beyond the interpretive strength of any single statistical test.

The findings of the present study diverge from several previously published pooled analyses. Cullinane et al. [11], in a meta-analysis of eight retrospective cohorts (1,586 patients), reported that lower pretreatment NLR values were associated with a higher likelihood of achieving pCR (OR 1.83; 95% CI 1.15–2.91). Similar observations were supported by a broader pooled analysis of circulating biomarkers, demonstrating a pooled OR of 1.66 (95% CI 1.32–2.09; *P <* 0.001) [12].

Regarding PLR, a large 2025 meta-analysis encompassing 24 studies and 7,557 patients found that elevated PLR correlated not only with a reduced probability of pCR (HR 1.51; 95% CI 1.24–1.84; *P <* 0.0001) but also with less favorable long-term outcomes [13]. Additionally, cohort studies by Yang et al. (95 patients) [14] and Kaytaz Tekyol et al. (150 patients) [15] similarly demonstrated significant relationships between inflammatory indices and pCR status.

However, three main factors may explain the divergent results observed in the current series compared to these aggregate analyses.

First, molecular subtype distribution significantly contributes to heterogeneity between study cohorts. The biological rationale for an association between NLR or SII and pCR is strongest in tumor subtypes where the immune microenvironment directly influences chemotherapy responsiveness, particularly TNBC and HER2-enriched breast cancers [11, 15]. In cohorts enriched with these immunologically active subtypes, peripheral inflammatory indices may partly reflect a systemic correlate of intratumoral immune activation, thereby corresponding to enhanced cytotoxic treatment sensitivity.

In the present cohort, however, TNBC and HER2-enriched tumors constituted approximately 37% of the study population, while luminal subtypes comprised the majority of cases (approximately 63%). Compared to TNBC and HER2-positive disease, luminal tumors generally exhibit lower chemosensitivity, reduced baseline immune infiltration, and mechanisms of pathological response less dependent on immune effector activity. Consequently, combining these four biologically distinct molecular populations into a single aggregate analysis without subtype-specific stratification may dilute potential subtype-driven immune associations, allowing the predominantly null relationships observed in luminal tumors to dominate the overall result.

A second, frequently underrecognized factor relates to the distributional range of inflammatory index values within individual cohorts. Thresholds reported as discriminatory in external studies, such as NLR ≥2.74 for HER2-negative patients described by Bae et al. [16] or the SII cut-off of ≥547 proposed by Jiang et al. [10], are generally designed to identify patients at the upper end of inflammatory distributions. In these patients, systemic immunosuppressive burden is presumed to be greatest and most likely to impair effective anti-tumor immune activity.

In the current cohort, however, the median NLR was 2.08 (IQR 1.59–2.95), and the median SII was 584.4. Both indices demonstrated relatively compact distributions centered below or near the externally derived threshold values. When the majority of patients fall within low-to-moderate inflammatory ranges, the discriminatory performance of any single cut-off becomes inherently limited. Population-level variability in baseline hematological characteristics, influenced by ethnic background, dietary patterns, and comorbidity profiles, may therefore represent an important yet often underappreciated contributor to the inter-institutional inconsistency observed throughout this field of research.

Third, the assumption that peripheral inflammatory ratios accurately reflect intratumoral immune competence remains biologically imperfect. Tumor-infiltrating lymphocytes (TILs) are well-established predictors of pCR in both TNBC and HER2-positive breast cancer [17, 18]. However, the circulating lymphocyte count, which contributes to indices such as NLR and SII, is influenced by numerous factors beyond tumor-related immunity, including hematopoietic reserve, redistribution of immune cells to peripheral tissues, concurrent inflammatory conditions, and systemic stress responses.

Accordingly, NLR represents a mathematical relationship between circulating immune cell fractions rather than a direct surrogate for intratumoral TIL density. In patients with high stromal TIL infiltration—a subgroup generally considered more responsive to immunogenic chemotherapy—the dissociation between peripheral inflammatory ratios and local immune effector activity may become even more pronounced. This biological disconnect may paradoxically weaken the very association these indices are theoretically expected to capture.

A significant methodological strength of this study, compared to many previous investigations, was the inclusion of the full ordinal RCB spectrum rather than limiting the analysis to binary pCR classification alone. Dichotomous pCR-based categorization inevitably reduces the information captured regarding residual tumor burden and may obscure associations distributed across the continuum from RCB-I to RCB-III rather than concentrated exclusively at the pCR threshold. To directly address this limitation, inflammatory indices were analyzed across all four RCB strata using the Kruskal–Wallis test. The resulting H statistics ranged from 0.130 to 0.580, with all corresponding *P*-values remaining above 0.90.

Among the evaluated indices, the SII exhibited its highest median value within the RCB-I subgroup (median 711.4). This pattern suggests random variation rather than a biologically coherent gradient associated with increasing residual disease burden. While the multicenter validation study by Yau et al. [3] established the prognostic significance of the RCB system itself, the present findings indicate that, within a mixed-subtype breast cancer cohort, pretreatment systemic inflammatory indices do not reliably predict a patient’s position along this prognostic continuum.

Nevertheless, an important nuance warrants acknowledgment. Previous studies, such as that by Yang et al. [14], have suggested that composite nomogram models integrating inflammatory indices with clinicopathological variables may offer incremental predictive value beyond that achieved by individual inflammatory markers alone. The analytical framework of the present study was designed to evaluate each inflammatory index as an independent, standalone predictor, reflecting common interpretation in routine clinical practice; thus, it does not directly address the utility of integrated models.

Accordingly, whether indices such as NLR or SII contribute additional predictive utility when incorporated into integrated multivariable models remains an open question. The current data neither support nor exclude such a potential role.

Several limitations should be considered when interpreting these findings. First, the retrospective, single-center design restricts generalizability and introduces the possibility of selection bias. Pre-analytical timing also represents a potentially significant source of variability. In instances where blood samples were obtained shortly after diagnostic core biopsy, biopsy-related inflammatory responses may have transiently increased neutrophil counts and reduced circulating lymphocyte levels, thereby introducing variability into inflammatory indices that may not accurately reflect a patient’s baseline immune status.

Another relevant limitation pertains to treatment heterogeneity. The study population included a range of neoadjuvant chemotherapy strategies, such as anthracycline-based monotherapy, sequential taxane administration, dual HER2-targeted blockade in HER2-positive disease, and carboplatin-containing regimens in TNBC. These regimen-specific differences were not incorporated into the multivariable regression model, despite the possibility that variations in treatment immunogenicity could influence the relationship between pretreatment immune status and pathological response. Detailed regimen-level information—including HER2-targeted therapy, platinum and anthracycline/taxane exposure, the number of cycles delivered, and treatment completion—was not uniformly retrievable from retrospective records and, therefore, could not be incorporated as covariates or used for regimen-stratified analysis. Given that the treatment regimen is strongly associated with both molecular subtype and pCR, residual confounding by treatment cannot be excluded, an acknowledged significant limitation.

Furthermore, subtype-specific analyses were not predefined and thus not performed. The available sample size did not provide sufficient statistical power for adequately controlled comparisons within individual molecular subgroups. More generally, with 41 pCR events and correspondingly wide confidence intervals, the study had limited power to detect small biomarker effects. Therefore, the null findings should be interpreted as the absence of a demonstrable association in this cohort rather than as definitive proof of no effect. Additionally, the Ki-67 threshold of 14% applied here reflects the St. Gallen convention in use during the study period; contemporary reporting increasingly favors laboratory-specific or continuous Ki-67 values, and the present dichotomous cutoff may not align with current practice. Finally, tissue-level immune biomarkers, including stromal TIL scores, programmed death-ligand 1 (PD-L1) expression, and lymphocyte subset phenotyping, were unavailable. Consequently, integrated analyses combining peripheral inflammatory markers with tumor microenvironment-related immune parameters, increasingly recommended in contemporary translational oncology research, could not be undertaken [17].

## Conclusion

Collectively, the findings from this retrospective cohort of 110 patients consistently indicate that pretreatment NLR, PLR, LMR, and SII values, all calculable from routine complete blood count parameters without additional cost, did not demonstrate meaningful predictive value for pathological response to neoadjuvant chemotherapy in this single-center, mixed-subtype cohort. Across all analytical approaches applied in the present study, these inflammatory indices consistently yielded either null or clinically negligible associations with treatment response.

In contrast, the Ki-67 proliferative index retained strong and independent predictive significance, with highly proliferative tumors demonstrating more than a sevenfold increased likelihood of achieving pCR. This observation further supports the concept that tumor-intrinsic biological characteristics remain the primary determinants of chemosensitivity in molecularly heterogeneous NACT populations. These findings do not support the routine clinical use of NLR, PLR, LMR, or SII as standalone pretreatment predictive biomarkers in this setting. Given the modest sample size, the mixed-subtype composition, and the absence of external validation or subtype-specific analysis, these results are best interpreted as the absence of a detectable association within the present study population rather than as definitive evidence that these indices lack predictive value in breast cancer more broadly.

Future progress in this field will likely depend on prospective study designs incorporating predefined molecular subtype stratification, standardized pre-analytical protocols capable of minimizing biopsy-related hematological variability, and integrated assessment of circulating inflammatory markers alongside tissue-level immune biomarkers. Under such controlled methodological conditions, the potential context-dependent predictive contribution of inflammatory indices, if present, may be more accurately defined.

## Supplemental data

**Table S1:** Cross-tabulation of molecular subtype by ER, PR, HER2, and Ki-67 status (N = 110)

**Molecular subtype**	***n***	**ER +**	**ER –**	**PR +**	**PR –**	**HER2 +**	**HER2 –**	**Ki-67** ≥**14%**	**Ki-67 <14%**
Luminal A	28	28	0	16	12	0	28	0	28
Luminal B	41	41	0	33	8	24	17	38	3
HER2-enriched	12	0	12	0	12	12	0	8	4
Triple-negative	29	0	29	0	29	0	29	21	8

Subtypes were assigned using the St. Gallen surrogate definitions, which were applied to the dichotomized immunohistochemical categories (ER and PR positive at ≥1%; HER2 positive by IHC 3+ or IHC 2+/ISH amplified; Ki-67 threshold 14%). All Luminal A tumors are hormone-receptor positive, HER2 negative, and Ki-67 <14%. HER2-enriched tumors are hormone-receptor negative and HER2 positive. Triple-negative tumors are negative for ER, PR, and HER2. This classification is fully consistent with the subtype definitions stated in the Methods. **Abbreviations**: ER, estrogen receptor; HER2, human epidermal growth factor receptor 2; IHC, immunohistochemistry; ISH, in situ hybridization; PR, progesterone receptor.

**Table S2:** Sensitivity multivariable logistic regression for pathological complete response, additionally including LMR (N = 110)

**Variable**	**OR**	**95 % CI**	**Wald Z**	***P* value**
NLR (per unit)	1.174	0.825–1.672	0.891	0.373
PLR (per unit)	1.002	0.996–1.008	0.632	0.528
SII (per unit)	0.999	0.998–1.001	−0.965	0.335
LMR (per unit)	0.930	0.760–1.150	−0.628	0.530
HER2-positive	2.383	0.897–6.333	1.741	0.082
Ki-67 ≥14% (ref: <14%)	7.327	2.267–23.679	3.328	0.001 *
Histological grade 3	1.433	0.500–4.107	0.670	0.503
ER-negative	1.654	0.605–4.518	0.981	0.327
Age (per year)	0.980	0.942–1.019	−1.010	0.313

*P < 0.05. This sensitivity model adds the lymphocyte-to-monocyte ratio (LMR) to the eight covariates of the primary model (Table 4); all nine covariates were entered simultaneously. Reference categories were HER2-negative, Ki-67 <14%, Grade 1–2, and ER-positive. LMR was not associated with pCR, and Ki-67 >=14% remained the only significant independent predictor (model c-statistic 0.79). A complete-case analysis was performed (N = 110). Abbreviations: CI, confidence interval; ER, estrogen receptor; HER2, human epidermal growth factor receptor 2; LMR, lymphocyte-to-monocyte ratio; NLR, neutrophil-to-lymphocyte ratio; OR, odds ratio; pCR, pathological complete response; PLR, platelet-to-lymphocyte ratio; SII, systemic immune-inflammation index.


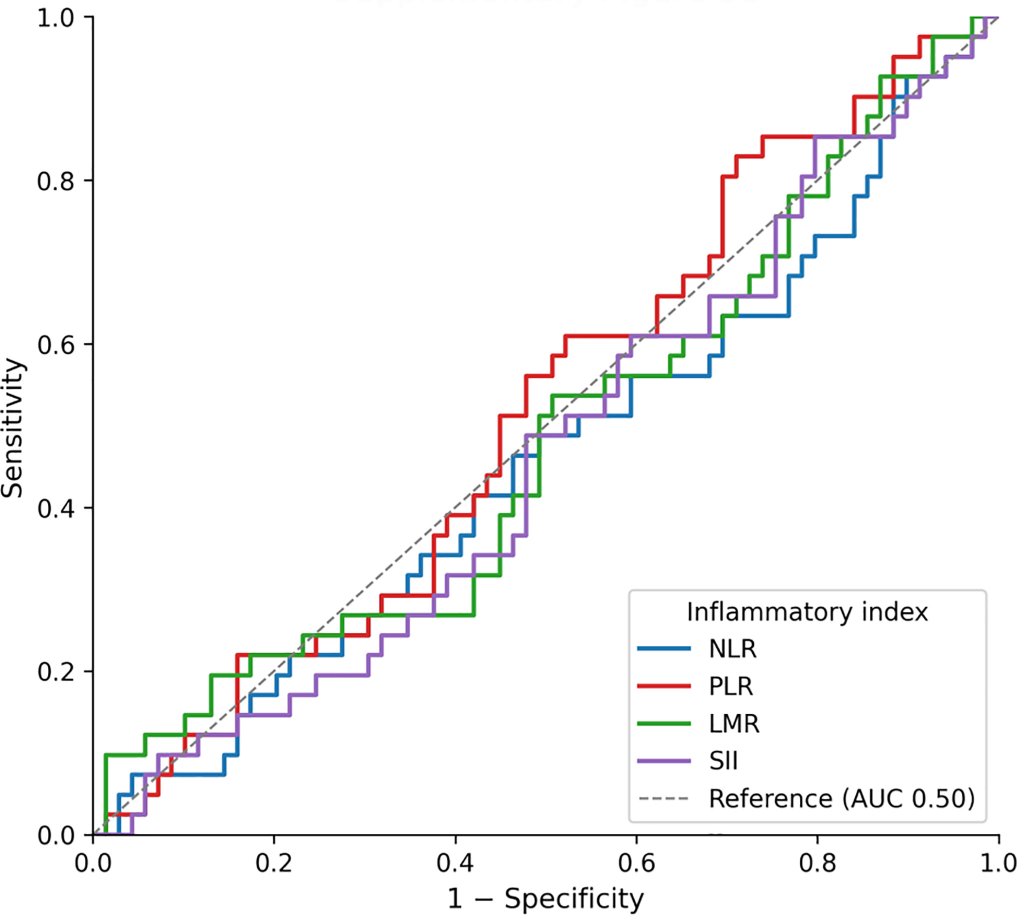


**Figure S1. ROC curves for pre-treatment inflammatory indices as classifiers of pathological complete response.** ROC curves show the discriminatory performance of NLR, PLR, LMR, and SII for predicting pCR after neoadjuvant chemotherapy in the full cohort (N = 110). All four curves closely followed the diagonal no-discrimination reference line, indicating poor classifier performance. Bootstrap-derived AUC values with 95% confidence intervals are reported in Table 5. These findings support the lack of clinically meaningful predictive value of pre-treatment inflammatory indices for pCR in this cohort. **Abbreviations**: AUC, area under the curve; CI, confidence interval; LMR, lymphocyte-to-monocyte ratio; NLR, neutrophil-to-lymphocyte ratio; pCR, pathological complete response; PLR, platelet-to-lymphocyte ratio; ROC, receiver operating characteristic; SII, systemic immune-inflammation index.
